# Neuroendocrine Factors in Melanoma Pathogenesis

**DOI:** 10.3390/cancers13092277

**Published:** 2021-05-10

**Authors:** Cristian Scheau, Carmen Draghici, Mihaela Adriana Ilie, Mihai Lupu, Iulia Solomon, Mircea Tampa, Simona Roxana Georgescu, Ana Caruntu, Carolina Constantin, Monica Neagu, Constantin Caruntu

**Affiliations:** 1 Department of Physiology, “Carol Davila” University of Medicine and Pharmacy, 050474 Bucharest, Romania; cristian.scheau@umfcd.ro (C.S.); costin.caruntu@gmail.com (C.C.); 2 Dermatology Research Laboratory, “Carol Davila” University of Medicine and Pharmacy, 050474 Bucharest, Romania; carmen.draghici3@gmail.com (C.D.); mihaelaadriana2005@yahoo.com (M.A.I.); lupu.g.mihai@gmail.com (M.L.); solomon.iulia.si@gmail.com (I.S.); 3 Department of Dermatology, “Carol Davila” University of Medicine and Pharmacy, 050474 Bucharest, Romania; tampa_mircea@yahoo.com (M.T.); simonaroxanageorgescu@yahoo.com (S.R.G.); 4 Department of Oral and Maxillofacial Surgery, “Carol Davila” Central Military Emergency Hospital, 010825 Bucharest, Romania; 5 Department of Oral and Maxillofacial Surgery, Faculty of Dental Medicine, “Titu Maiorescu” University, 031593 Bucharest, Romania; 6 Immunology Department, Victor Babes National Institute of Pathology, 050096 Bucharest, Romania; caroconstantin@gmail.com (C.C.); neagu.monica@gmail.com (M.N.); 7 Department of Pathology, Colentina University Hospital, 020125 Bucharest, Romania; 8 Faculty of Biology, University of Bucharest, 076201 Bucharest, Romania; 9 Department of Dermatology, “Prof. N. Paulescu” National Institute of Diabetes, Nutrition and Metabolic Diseases, 011233 Bucharest, Romania

**Keywords:** melanoma, neurotransmitters, neurohormones, neuropeptides, stress

## Abstract

**Simple Summary:**

Melanoma is a very aggressive and fatal malignant tumor. While curable if diagnosed in its early stages, advanced melanoma, despite the complex therapeutic approaches, is associated with one of the highest mortality rates. Hence, more and more studies have focused on mechanisms that may contribute to melanoma development and progression. Various studies suggest a role played by neuroendocrine factors which can act directly on tumor cells, modulating their proliferation and metastasis capability, or indirectly through immune or inflammatory processes that impact disease progression. However, there are still multiple areas to explore and numerous unknown features to uncover. A detailed exploration of the mechanisms by which neuroendocrine factors can influence the clinical course of the disease could open up new areas of biomedical research and may lead to the development of new therapeutic approaches in melanoma.

**Abstract:**

Melanoma is one of the most aggressive skin cancers with a sharp rise in incidence in the last decades, especially in young people. Recognized as a significant public health issue, melanoma is studied with increasing interest as new discoveries in molecular signaling and receptor modulation unlock innovative treatment options. Stress exposure is recognized as an important component in the immune-inflammatory interplay that can alter the progression of melanoma by regulating the release of neuroendocrine factors. Various neurotransmitters, such as catecholamines, glutamate, serotonin, or cannabinoids have also been assessed in experimental studies for their involvement in the biology of melanoma. Alpha-MSH and other neurohormones, as well as neuropeptides including substance P, CGRP, enkephalin, beta-endorphin, and even cellular and molecular agents (mast cells and nitric oxide, respectively), have all been implicated as potential factors in the development, growth, invasion, and dissemination of melanoma in a variety of in vitro and in vivo studies. In this review, we provide an overview of current evidence regarding the intricate effects of neuroendocrine factors in melanoma, including data reported in recent clinical trials, exploring the mechanisms involved, signaling pathways, and the recorded range of effects.

## 1. Introduction

Melanoma is one of the most fatal malignant tumors and is responsible for the majority of deaths associated with skin tumors worldwide. Moreover, it has recorded a sharp rise in incidence during the last decades, especially in young people [1,2,3]. Hence, melanoma is a significant public health problem [4] and an increasing number of studies are investigating the mechanisms involved in melanoma development.

Originating from melanocytes, melanoma has an established genetic component [5]. However, several other factors, such as ultraviolet radiation (UV), chronic inflammation, or impairment of immune-modulatory pathways can play a significant role in its initiation and progression [6,7,8,9,10,11,12,13,14].

Moreover, the results of numerous studies have suggested a potential impact of a variety of signaling agents such as hormones, neurotransmitters, neuropeptides in the evolution of melanoma [15,16,17,18].

**Figure 1:**
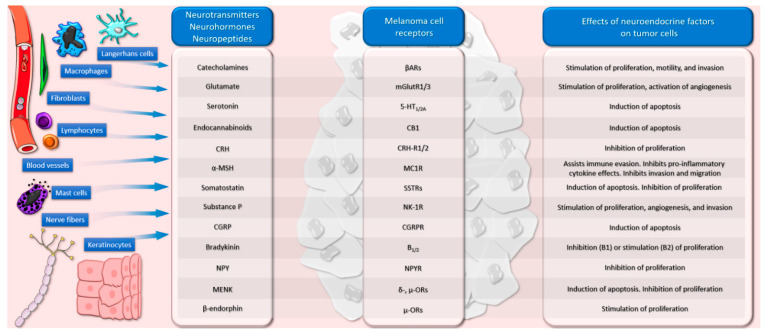
Neuroendocrine factors produced in different compartments of the skin and their most relevant effects on melanoma cells via specific receptors.

In this review, we provide an overview of current evidence regarding the connection between the neuroendocrine factors and melanoma, also focusing on the cellular and molecular neuro-immune interactions possibly involved (see Figure 1).

**Figure 2:**
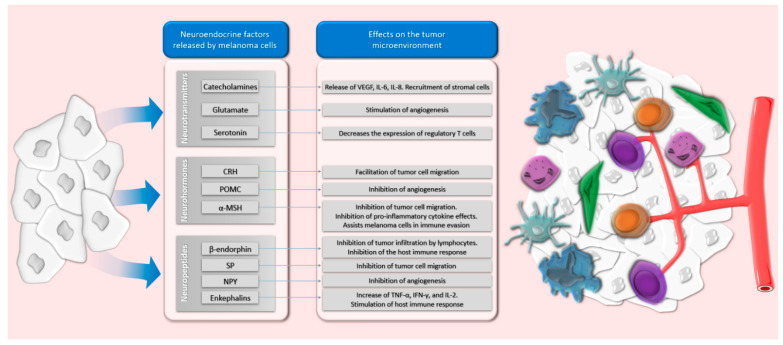
Neuroendocrine factors released by melanoma cells and their effects on the tumor microenvironment.

Moreover, melanoma cells are able to secrete some of these neuroendocrine factors, and subsequently impact the tumor microenvironment (see Figure 2).

Neuroendocrine factors are important regulators of various cutaneous physiological and pathophysiological processes [19,20,21,22,23,24,25,26] and the investigation of their involvement in the complex mechanisms leading to melanoma development is of major interest in scientific research, being able to contribute to the identification of new targets for melanoma therapy.

## 2. Melanoma and Stress

Chronic stress is highlighted by various studies as one important and common cofactor not only in the initiation and progression of different skin disorders [25,26,27] but also in the evolution of melanoma [6,28]. Stressful occupations are reported to increase the risk of melanoma [6] and studies involving melanoma patients suggest the implication of psycho-emotional factors in disease progression [29,30,31,32,33,34,35,36].

Exposure to stress activates the hypothalamic–pituitary–adrenal axis, with consequent release of glucocorticoids from the adrenal cortex, and increases the activity of the sympathoadrenal system inducing the release of epinephrine from the adrenal medulla and norepinephrine (NE) from sympathetic nerve endings [37]. While studies have shown that melanoma cells in different disease stages have the potential to respond to stress hormones, this response may vary amongst different cell populations [38].

Kanno et al. showed that exposure to restraint stress in mice can induce pattern changes in B16 cell lung metastases, regarding both the number and size of metastatic colonies [39]. Surgical-stress induced to C57BL/6 mice prior to B16-BL6 melanoma cells inoculation was proven to significantly increase pulmonary metastases [40]. A correlation between social stress exposure, passive-reactive coping strategy, high serum corticosterone, and an increased number of B16-F10 melanoma pulmonary metastases has also been highlighted [41].

One possible link between stress and melanoma is through the immune-inflammatory processes that impact disease progression. Studies in psycho-neuro-immunology have shown that psychological stress can influence many aspects of the specific and innate cellular immune responses, mediated by the endocrine system through bidirectional interactions. Activation of the hypothalamic–pituitary–adrenal axis and sympathoadrenal system leading to an increase in stress hormone levels, has been shown to induce immune dysregulation, with overproduction of proinflammatory cytokines, such as interleukin (IL)-1, tumor necrosis factor-α (TNF-α), and IL-6 [42,43,44,45]. In its turn, inflammation has been associated with the development of numerous types of cancer, including melanoma [13]. Hence, stress could act as a cofactor in the inflammatory promotion of tumor growth and progression.

Moreover, stress-induced changes in cellular immune responses lead to a decrease in specific T cell and natural killer (NK) cell responses, impairing tumor immune surveillance [42,46].

Other research has also emphasized the connection between stress-induced neuroendocrine factors, proinflammatory cytokines, and the metastatic potential of melanoma. Using a B16F10 melanoma metastasis mouse model, it was shown that increased levels of stress-related hormones are associated with the promotion of lung and liver metastases [47]. In in vitro studies using the B16F10 melanoma cell line, high levels of corticosterone and NE induced an increased expression and secretion of IL-6. This cytokine has an important role in liver metastatic invasion. IL-6 can also generate an increased expression of proteins such as B-cell lymphoma (Bcl)-2, Bcl-xl, myeloid leukemia cell (Mcl)-1, survivin, and X-linked inhibitor of apoptosis protein (XIAP), providing cancerous cells with an important survival mechanism [47]. Corticosterone and NE may generate transcriptional upregulation of the IL-6 gene with changes in DNA binding activity of nuclear factor-kB (NF-κB), activator protein-1 (AP-1), and nuclear factor for IL-6 [47].

Furthermore, stress can also have a different effect on tumor progression, independent of immune disturbances. Stress-induced activation of sympathoadrenal system can promote tumor progression by modulating the expression of proangiogenic and prometastatic factors [48].

A recent study by Ben-Shaanan et al. has revealed a more complex connection between the psychological state of a patient and tumor progression. The authors have shown that activation of the reward system inhibits melanoma growth on a B16 murine model, which is correlated with a decrease in NE levels with consequent effects on myeloid-derived suppressor cells [49].

It is thus apparent that chronic psychological or environmental stress, through the action of different neuroendocrine factors and cellular and molecular neuro-immune interactions, is involved not only in melanoma initiation but also in its progression and metastasis.

## 3. Neurotransmitters and Melanoma

Neurotransmitters are a group of endogenous substances that facilitate signal transmission from one neuron to another, to muscle, glandular or other target tissues, playing critical roles in regulating of numerous physiological and pathophysiological processes [50]. The impact of several neurotransmitters in melanoma has been evaluated in clinical and experimental studies.

### 3.1. Catecholamines

Various studies have indicated a possible link between modulation of sympathoadrenal system activity and evolution of melanoma. Accumulating evidence indicates that sympathetic stimulation may favor tumor progression and, conversely, reducing the activity of the sympathoadrenal system exerts inhibitory effects on melanoma. For example, in a melanoma mouse model using B16F10 melanoma cells, Horvathova et al. found that chemical sympathectomy induces complex changes in the tumor microenvironment, significantly attenuating melanoma growth [51]. Additionally, recent studies have shown an association between Parkinson’s disease (PD) and melanoma, the presence of one of these diseases increasing the chance of developing the other [52]; while the mechanisms connecting these two diseases have not been fully uncovered, it was suggested that the loss of dopaminergic neurons may favor the onset of melanoma, which occurs nearly four times more frequently in patients with PD [53]. Genetic similarities have also been described between melanoma and PD, further consolidating the linkage in the pathophysiology of the two conditions [54].

The presence of adrenergic receptors was emphasized in different types of solid tumors and it has been shown that activation of adrenergic receptors by catecholamines can stimulate tumor cell proliferation, activate antiapoptotic processes, induce angiogenesis, and favor metastasis [17,55]. The role of adrenergic receptors in melanoma was brought to attention by several research groups [16]. Alpha (α)-adrenoceptors and beta (β)-adrenoceptors are expressed in both melanoma tissue samples and various melanoma cell lines [38,56,57,58]. However, adrenergic receptor expression and melanoma cell response to adrenergic stimulation are dependent on both the receptor and the cell type [59].

**Figure 3:**
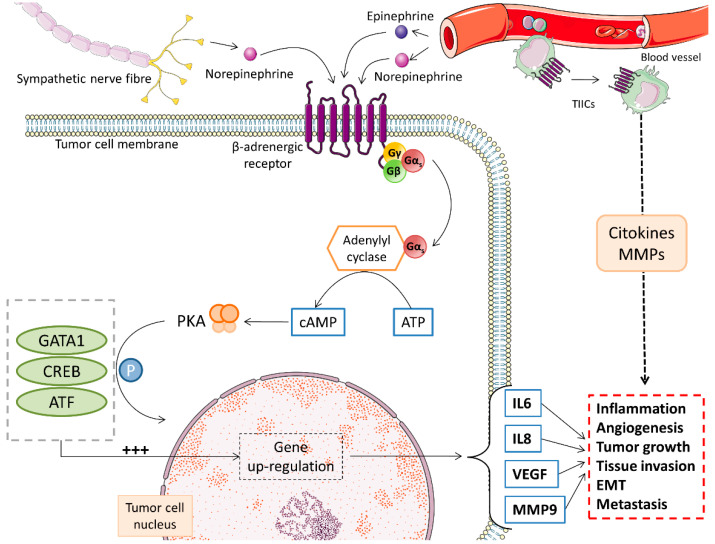
Role of β-adrenergic modulatory mechanisms in tumor development and progression. Release of catecholamines in the tumor tissue associated with activation of sympathoadrenal system, stimulates β-adrenergic receptors on melanoma and tumor microenvironment cells. In tumor cells it further induces an increase of cAMP formation which, in its turn, activates multiple intracellular signaling pathways, such as GATA1, CREB and ATF, initiating protumorigenic mechanisms. Adrenergic stimulation of tumor infiltrating immune cells (TIICs) increases the production of proinflammatory cytokines and matrix metalloproteinases (MMPs), also favoring tumor growth, invasion and metastasis.

The β-adrenergic system seems to play a key role in the sympathoadrenal interplay with melanoma. Various studies have demonstrated the presence of functional β-adrenergic receptors in melanoma cells and also in the tumor microenvironment. As with other solid tumors, activation of β-adrenergic receptors by catecholamines in melanoma is responsible for initiating protumorigenic pathways by stimulating cellular proliferation and motility, immune system regulation, antiapoptosis, epithelial–mesenchymal transition, invasion, neo-angiogenesis, and the occurrence of metastasis [16,55,60] (see Figure 3).

There are many studies investigating β1 and β2 adrenergic receptor expression in melanoma cell lines. Researchers have focused on three human melanoma cell lines: C8161, 1174MEL, and Me18105 and the investigations concluded that NE can escalate tumor progression, by stimulating the secretion of factors involved in angiogenesis and metastasis, but also to upregulate vascular endothelial growth factor (VEGF), IL-8, and IL-6 in C8161 cells, and to a lesser extent in Me18105 and 1174MEL cells. These first three cytokines play proangiogenic, chemotactic, or autocrine stimulating roles and are closely related to melanoma progression since their production by melanoma cells is elevated in advanced tumor stages [38,61].

The underlying mechanisms of NE-induced upregulation of VEGF, IL-8, and IL-6 expression are not yet fully deciphered. However, these factors have been involved in several processes associated with tumor progression in melanoma, including angiogenesis [62]. For example, VEGF released by melanoma cells is an important mediator of neovascularization and is a marker of progression [63,64]. IL-6 levels have been associated with advanced disease states. Furthermore, IL-8 and IL-6 are released into the supernatant of highly invasive melanoma cell cultures [65,66,67]. Some factors are involved in angiogenesis either by direct stimulation of proliferation and differentiation of endothelial cells or by induction of extra factors production in other cells [65].

Studies on melanoma cell lines have demonstrated that stimulation by catecholamines activates p42/p44 and p38 mitogen-activated protein kinases (MAPKs) pathways, which are responsible for growth, survival, and metastatic cancer cell responses, along with increased production of matrix metalloproteinase (MMP)-2 and 9, IL-6, IL-8, and VEGF [17,38,58]. These factors have a strong protumorigenic effect [68,69]. Additionally, catecholamines are responsible for increasing melanocyte motility in both primary and metastatic melanoma cell lines [58].

The same research groups have highlighted the possible regulatory action of β-adrenergic receptors on the protumorigenic response generated by the melanoma microenvironment. Thus, via the β-adrenergic system, soluble mediators are produced to activate the cells that belong to the melanoma microenvironment, in turn, generating new protumorigenic mediators, thereby maintaining stimulation of melanoma growth and, implicitly, the occurrence of metastases [17,38,58].

Furthermore, the presence of β2-adrenoreceptors was associated with tumor ulceration, higher disease stage, and increased tumor size [70]. Another important protumorigenic role is given by the correlation between melanoma cells and the constituent cells of the microenvironment, through stimulation of β2 and β3 adrenergic receptors by catecholamines [70,71,72,73,74]

Studies have demonstrated the presence of β3-adrenergic receptors in B16F10 cells, and also their involvement in regulating the angiogenic response to hypoxia [17,38,47,58]. Thus, blocking β3-adrenergic receptors using selective antagonists led to the reduction of tumor cell proliferation and increased apoptosis of these cells. It has also been shown that inhibition of these receptors has no direct effect on the production of angiogenic factors by neoplastic cells. It should be taken into consideration that usage of selective β3-adrenergic receptor blockers leading to decreased vascular proliferation might stand at the basis of tumor involution [71].

Other studies have demonstrated the presence of β3-adrenergic receptors in both cutaneous melanoma cells and other cell populations responsible for the melanoma microenvironment, such as fibroblasts, endothelial cells, and macrophages [72]. These receptors are, however, expressed differently in melanocytic nevi as opposed to primary or metastatic melanomas, with significant upregulation in the latter [73].

It appears that β-adrenergic receptors are expressed differently in the constituent cells of the melanoma microenvironment (macrophages, fibroblasts and endothelial cells), with a majority of β3-adrenergic receptors at this level. This may emphasize the involvement of β3-adrenergic receptors in maintaining tumor progression through their action on the constituent cells of the microenvironment. Other research has shown that melanoma cells under NE stimulation can lead to the recruitment of associated fibroblasts. At the cellular level, NE acts on the β3-adrenergic receptors expressed by fibroblasts, releasing protumorigenic cytokines: IL-8, IL-6, VEGF, and fibroblast-2 growth factor (FGF-2) [58,73,75,76,77]. These fibroblasts can increase their basal expression of alpha-smooth muscle actin under NE action by generating myofibroblasts, stimulating melanocytic cell motility and causing neo-angiogenesis [75,76,78], thus favoring metastatic behavior [58]. Additionally, β3-adrenergic receptors mediate the decrease of regulatory T cells and myeloid-derived suppressor cell populations in B16F10 melanoma tumors in mice, facilitating immune tolerance [79]. Another effect of NE is the induction of elevated levels of CD20 and CD33 by the expression of stem-like traits in melanoma cells [80,81].

According to the above information, β-adrenergic receptors play an important role in tumor proliferation and are involved in all stages of tumorigenesis. Thus, understanding the role of the β-adrenergic system in melanoma development can lead to new therapeutic approaches. The stimulation of neoplastic cell development seems to be a mechanism dependent on the status and subtype of β-adrenergic receptors expressed by each tumor. There is evidence that modulation and inhibition of the biological effects associated with the activation of β-adrenergic receptors can be achieved through the use of different types of beta-blocker agents [82]. According to several studies, β-adrenergic receptor blockade can slow melanoma progression and lead to an important decrease in the risk of tumor recurrence and mortality [83,84], improving the clinical outcomes in this type of cancer [85]. Moreover, research regarding the use of immunotherapy in melanoma has shown improved survival rates under treatment with beta-blockers in association with dual alfa-programmed death receptor-1 and high dose IL-2 therapy with action on β2-adrenoreceptors [86,87]. Additionally, another important finding is the influence of nitric oxide (NO) on β3-adrenoreceptors. The study of Dal Monte et al. showed that the effects of β3-adrenoreceptors activation/blockade depend on inducible nitric oxide synthase (iNOS) inhibition/activation. Treatments that stimulate NO production inducing an increased expression of iNOS also determine the blockage of β3-adrenoreceptors, while therapies that decrease NO production reducing iNOS expression, in turn activate β3-adrenoreceptors [88]. A detailed description of the impact of NO and different types of NOS on melanoma will be presented in the following sections.

De Georgi et al. conducted a retrospective study including 121 patients diagnosed with thick melanoma that were under treatment with beta-blockers, showing an important reduction of melanoma progression risk. The same group studied the effect of beta-blockers on other patients with cutaneous melanomas [84,85], arriving at similar conclusions. These results were confirmed by Lemeshow et al., in a study on 4179 patients diagnosed with melanoma in Denmark, pointing out that there was an increase in the survival time of patients who received beta-blockers. Other studies performed by different authors outlined the same information, suggesting that this class of medication can be a good addition to melanoma treatment strategy [83]. These observations were also tested in vitro, on A375 human melanoma cell lines by attempting to attenuate the proliferative effect of NE with several specific inhibitors, such as yohimbine, propranolol, and phentolamine, observing mixed and dose-dependent results [89].

However, some studies do not confirm the positive role of beta-blockers on melanoma evolution. McCourt et al. studied the effect of beta-blockers on a group of patients with melanoma from the UK, and revealed that these drugs could not be associated with a reduced risk of melanoma [90], a conclusion also supported by a study published by Livingstone et al. in 2013 [91].

A possible explanation regarding these divergent results was suggested by the study of Wrobel and Le Gal which outlined the effect of non-cardioselective beta-blockers such as propranolol that can inhibit both β1 and β2 adrenoreceptors in comparison with cardioselective beta-blockers that act only on β1-adrenoreceptors. Propranolol is considered to have an important role in the inhibition of melanoma growth by modulating angiogenesis, tumor proliferation, and cell survival [92]. Studies have shown that propranolol induces a decrease in tumor development and angiogenesis, both in primary tumors and metastatic ones, and also reduces the myeloid cell infiltration of primary tumors [93,94,95].

Other studies have also described antitumorigenic roles of catecholamines. In an experimental study on a subcutaneous B16F10 melanoma model in female mice, Pederson et al. have outlined that regular exercise significantly reduces tumor incidence and growth. Exercise is associated with an acute mobilization of NK cells induced by increased epinephrine, an effect dependent on β-adrenergic receptors activation. Blocking of β-adrenergic signaling was shown to reduce the epinephrine-induced mobilization of NK cells and obliterated the effect of exercise on tumor incidence and growth. Moreover, it reduced the infiltration of NK cells into tumors. Exercise also induced a significant increase in IL-6 serum level which was associated with an epinephrine-induced mobilization of IL-6Rα-positive NK cells. Furthermore, anti-IL-6 antibodies reduced the tumor NK cell infiltration. The results of this interesting research suggest that regular exercises can inhibit tumor progression through direct regulation of NK cells with the participation of epinephrine, which mobilizes NK cells to the circulation, but also with the IL-6 dependent redistribution of these cells into the tumor [96].

SK-Mel 23 melanoma cells express low-affinity α1-adrenoreceptors which are coupled to Gq proteins that produce a subsequent calcium influx when stimulated. Activation by catecholamines or other agonists can exhibit the biological effect of proliferation decrease and increase in tyrosinase activity via involvement of p38 and extracellular signal-regulated kinases (ERKs) [28,56,97].

Even if the role of the adrenergic system is not yet fully clarified, the data presented suggest that catecholamines may be involved in the promotion of the aggressive potential of melanoma cells, by interacting with specific receptors. Intervention on these receptors or impacting the catecholomine production during the stress response, through pharmacological treatment with adrenoreceptor blocking agents or social and psychological support, must be investigated as complementary approaches in the treatment of melanoma [38]. In this general context, it is clear that more studies regarding the effect of different types of beta-blocking agents on survival rates in melanoma are needed.

### 3.2. Glutamate

Glutamate is the most abundant excitatory neurotransmitter in the human central nervous system, where it plays a critical role in intercellular communication [98]. The glutamate receptor family is divided into two major groups: ionotropic glutamate receptors (iGluR) and metabotropic glutamate receptors (mGluR). The former group is divided into four subtypes: N-methyl-D-aspartate (NMDA) receptors, α-amino-3-hydroxy-5-methyl-4-isoxazolepropionic acid (AMPA) receptors, kainate, and delta receptors, while the latter are subdivided into three groups of seven-transmembrane domain G protein-coupled receptors. In addition to the nervous system, the glutamate receptors are also expressed in non-neuronal tissues, being involved in the modulation of various normal or pathological processes [99].

The role of glutamate signaling in melanoma has been the subject of investigation for several studies, which reported that melanoma cells express different types of glutamate receptors, over 60% of human melanomas expressing metabotropic glutamate receptors, including mGluR1 and 5 [99,100,101].

In a complex study [102] using an insertional mutant mouse model predisposed to develop multiple melanomas, Pollock et al. have shown that mGluR1 was ectopically expressed in melanoma tumors, even if its expression was not detected in normal mouse melanocytes. A similar pattern was demonstrated in human cells, with expression of mGluR1 detected in melanoma cell lines and tumor tissue but not in normal melanocytes and benign nevi, suggesting its contribution to melanoma development. Using the same in vivo mouse model, it was shown that ectopic expression of mGluR1 in melanocytes plays a critical role in the onset of melanoma, being sufficient to induce the development of melanoma tumors [102]. Other research has reported that activation of every signaling pathway associated with mGluR1 is required for the development of melanoma [101]. In another experimental study, Ohtani et al. have shown that mGluR1 is essential, not only for the development of melanoma but also for tumor growth [103].

Moreover, since melanoma cells release significant amounts of glutamate, an autocrine loop could be taken into consideration [104]. A recent study showed that autocrine stimulation acts on melanoma cells, causing the activation of mGluR1 and subsequent signaling cascades, with increased expression of glutaminase that converts glutamine to glutamate [105]. Additionally, high levels of extracellular glutamate may be associated with glutamate ionotropic receptor NMDA type subunit 2A gene (GRIN2A) mutations, frequently found in melanoma tumors [106].

Further investigation of mGluRs in controlling melanoma development has led to the observation that the regulation of tumor cell viability must rely on a mechanism requiring receptor internalization. Gelb et al. observed that the inhibition of dynamin decreases the life span of melanoma cells expressing mGlu1R, therefore concluding that the model of glutamate receptor function in melanoma should be updated and refined [107].

Some studies revealed an aberrant expression of mGluR1 in a subset of human melanoma cell lines, suggesting its contribution to melanoma development. Murine studies have shown that activation of mGluR1 induces in vitro melanocytic transformation and in vivo spontaneous melanoma development [108]. Thus, genetic modulation in the expression of mGluR1 by siRNA or alterations in the transmission of signals mediated by mGluR1 can cause cell proliferation and neoplastic progression [109]. In another study, ectopic overexpression of mGluR1 was deemed sufficient for neoplastic transformation, and a correlation between receptor density and tumor size was identified in vivo, explained by the higher concentration of IL-8 and VEGF generated under the influence of mGluR1 [110].

Another metabotropic receptor, mGluR3 was recently investigated for its role in tumorigenesis, and study data shows that mGluR3 may be involved in melanoma development by altering cAMP signaling [111].

The antiproliferative potential of glutamate antagonists has been investigated in several types of cancer including breast, colon, brain, and lung [112]. There are studies that incriminate the involvement of glutamate signaling in melanoma development through mGluRs and/or iGluRs, suggesting that they could be considered as novel targets for melanoma therapy.

Song et al. revealed that mGluR1 and NMDA receptor antagonists induce increased dendritic arborization and suppress the development and spreading of cancerous melanocytic cell lines. As an important aspect, augmented expression of microtubule-associated protein (MAP) 2a alongside the presence of either of the two aforementioned antagonists yields the potential of decreasing melanoma cells’ capacity for invasion and metastasizing [113].

Recently, ^131^I and ^211^At small-molecule radionuclides have been used to target mGluR1, inhibiting melanoma growth in B16F10 melanoma cells in mice and showing quick clearance after injection [114].

A study on mouse tumor models showed that mGLuR 2/3 antagonist LY341495 inhibits the growth of B16F10 melanoma cells via regulating the activity of Myeloid-Derived Suppressor Cells, thus reaffirming the role of glutamate signaling in suppressing immunity and facilitating tumor progression [115].

Furthermore, mouse melanoma cell proliferation was inhibited by blocking NMDA receptors with MK-801 and the effect was amplified when antiestrogens were associated; among all iGluRs, only the blocking of NMDA receptors was effective in controlling melanoma growth [116].

For some glutamate antagonists, the research has progressed rapidly. Clinical trials in patients with advanced melanomas showed that riluzole, a mGluR1 signaling inhibitor, can induce tumor regression through the inhibition of mitogen-activated protein kinase and phosphoinositide 3-kinase/protein kinase B [100,110,117]. Other studies analyzed the effects of the combination of riluzole and sorafenib for melanoma treatment and evidenced that this combination treatment is efficient in suppressing tumor proliferation both in vivo and in vitro [118]. Riluzole upregulates the expression of transforming growth factor-beta (TGF-β) signaling pathway genes and induces a noncanonical augmentation of Smad linker phosphorylation [119]. Additionally, riluzole causes an increased expression of p21 Waf1/Cip1 and p53 [120]. Dipeptide conjugates of riluzole have been developed in order to overcome the liver toxicity and variable exposure of riluzole, and the derivate FC-3423 was reported to strongly suppress melanoma growth in mice at a fraction of the molar dose of riluzole [121].

### 3.3. Serotonin

Serotonin, also known as 5-hydroxytryptamine (5-HT) is a monoamine neurotransmitter which plays an important modulatory role in multiple psychological and social behaviors [122]. The production of 5-HT is stimulated by exposure to light, and additional research on several cell lines has demonstrated that 5-HT is capable of inducing the synthesis of melanin by interacting with 5-HT_2A_ receptors [123,124].

Skin cells, including melanocytes, produce 5-HT [125] and immune and vascular systems located in the skin are targets for 5-HT bioregulation [126,127]. The capability of melanoma cells to synthesize and metabolize 5-HT has been documented and this ability was attributed to their neural crest origin [128,129].

The role of 5-HT receptors in melanoma has been investigated on several melanoma cell lines using a serotonergic derivative of quipazine with dual action, operating as an agonist and antagonist on various 5-HT receptors [130]. Thus, Menezes et al. demonstrated that 1-(1-Naphthyl) piperazine causes apoptosis in melanoma cells by inducing oxidative stress in a dose-dependent manner.

Another 5-HT derivate, N-dihydroxyphenil-acylserotonin was able to suppress melanin with no cytotoxicity on B16 melanoma cell lines via the inhibition of tyrosinase through the 5-hydroxyindole moiety [131].

In order to evaluate the role of 5-HT in tumor progression, Naimi-Akbar et al. have investigated the alterations of the serotonergic system in superficial spreading malignant melanoma as compared to dysplastic compound nevi and benign compound nevi [132]. The authors have performed an immunohistochemical analysis regarding the expression of 5-HT, serotonin transporter protein (SERT), and 5-HT receptors 5-HT_1A_ and 5-HT_2A_. They have revealed that tumor cells can express different serotonergic markers, melanoma cells being able to express both 5-HT_1A_ and 5-HT_2A_ receptors. Moreover, it was noticed a fading of the immunoreactivity for serotonergic markers, such as 5-HT_1A_ and SERT as the degree of atypia increased. Additional studies found overexpression of 5-HT_2B_ in uveal melanomas, promoting this protein as a potential therapeutic target [133].

5-HT receptor agonists have been explored as anti-melanoma agents. Tegaserod has proven effective in inducing apoptosis and limiting tumor and metastasis development in mice inoculated with B16F10 melanoma by decreasing the expression of CD4^+^CD25^+^ T cells and hindering the PI3K/Akt/mammalian target of the rapamycin (mTOR) signaling pathway [134].

There is also a different approach regarding the relationship between melanoma and the 5-HT system. In an experimental model Lebena et al. have demonstrated that mice inoculated with B16F10 melanoma cells express alterations of proinflammatory cytokines production and dysfunctions of the central monoaminergic system, with a decrease in dopaminergic activity and 5-HT turnover in several brain areas. These disturbances are linked to depressive-like behavior in these animals offering a possible explanation regarding the mechanisms underlying the relationship between depression and cancer [135].

Moreover, antidepressant drugs, such as fluoxetine, which is a selective 5-HT reuptake inhibitor (SSRI), may have an antitumoral effect owing to increased mitogen-induced T cell proliferation and may play a key role in regulating melanogenesis [136,137]. Sertraline, another SSRI, was shown to decrease growth, proliferation and cellular migration in murine and human melanoma cell lines in vitro (B16-F10 and A2058, respectively), through another mechanism, namely by acting on translationally controlled tumor protein [138]. On the other hand, desipramine, a tricyclic antidepressant that inhibits NE reuptake, may stimulate metastasis development and increased mortality rate in young animals, in correlation with increased levels of VEGF and MMP-9 in plasma. Desipramine also reduced primary cancerous cell growth in young animals, while in aged animals, both fluoxetine and desipramine determined primary tumor growth. A decrease in the production of IL-12p40 at the level of unstimulated desipramine-treated splenocytes obtained from young animals was observed, while low levels of IL-12p40 were seen in Con A-stimulated splenocytes from mice treated with desipramine and fluoxetine [139].

The cancer-inhibiting role of antidepressants was further established in a recent registry-based study which found that SSRIs and mixed antidepressants decrease the risk for melanoma development, most likely as a direct action of the drug and, to a lesser extent by the reduced exposure to UV of these patients [140]. Conversely, a retrospective study found that continuous SSRI usage correlated with lower survival in melanoma patients, compared to past use [141]. However, these antidepressants have different mechanisms of action and activities targeting the immune system that could explain their disparate effects on tumor growth and metastasis formation. Therefore, further studies are needed to uncover the complex interactions between 5-HT metabolism and the development of melanoma.

### 3.4. Cannabinoids

Cannabinoids represent a class of compounds that act on the endocannabinoid system, modulating the release of neurotransmitters in the brain [142].

Although cannabinoids are involved in cell proliferation control, there is little information about their role in human malignant melanoma [143]. Cannabinoids exhibit anti-inflammatory properties and their impact on the tumor inflammatory milieu was suggested as a potential action mechanism against the development of melanoma and other skin cancers [144].

Human melanoma cells express CB1 and CB2 cannabinoid receptors. Their activation leads to inhibition of growth, proliferation, angiogenesis, and metastasis, and to the enhancement of melanoma cell apoptosis in mice [145]. Moreover, a recent study performed on human melanoma cell lines has revealed that the CB1 receptor may act as a tumor-promoting signal in cutaneous melanoma [146].

Kenessey et al. demonstrated that administration of stable CB1 agonist, Arachidonyl-2’-chloroethylamide, ACEA, may inhibit liver colonization with human melanoma cells [147].

Recent findings suggest that anandamide (AEA), an endocannabinoid, can stimulate cytotoxicity in human melanoma cells. This mechanism implies the synthesis of cyclooxygenase-2 and lipoxygenase-derived products and activation of CB1. Moreover, lipid raft modulation, probably involving GPR55 activation, may play an important part in this process, possibly by regulating CB1 signaling through its intracellular trafficking, as demonstrated on alternate tumor models [143,148].

Cannabidiol exhibited antitumor effects in a recent study on a murine model implanted with B16F10 melanoma tumors, and improved the quality of life in mice, compared to the treatment with cisplatin, albeit not demonstrating the same effectiveness in increasing survivability [149].

WIN 55,212-2 (WIN), another cannabinoid agonist, exerts antiproliferative effects on human melanoma cells, which express mRNA and protein for CB1 and CB2 receptors. The membrane lipid raft complex-mediated antimitogenic effect of WIN suggests potential future therapies in melanoma treatment [150].

Novel therapies targeting autophagy, which may promote cancer cell death in chemotherapy-resistant melanomas, are of great interest, especially for BRAF/NRAS wild-type melanomas. Moreover, recent studies on mice suggest that Δ(9)-Tetrahydrocannabinol (THC) can activate noncanonical autophagy-mediated apoptosis of melanoma cells [151].

Bachari et al. conducted a systematic review including in vivo studies on the effects exerted by cannabinoids on melanoma cells and pointed out that cannabinoids decrease tumor proliferation and stimulate apoptosis and autophagy in melanoma cells. In addition, it seems that the use of a combination of two cannabinoids may be more efficient than the use of individual cannabinoids [152].

Despite their promising potential as anti-melanoma agents, there are reports of cannabinoids negatively impacting the melanoma reaction to immunotherapy. A retrospective study reported a significant decrease in the response rate to nivolumab in cannabis users with advanced melanoma and other malignancies [153]. Therefore, additional studies are needed to clarify the impact of cannabinoids in different areas of melanoma research.

A panel of studies exploring the involvement of neurotransmitters in the development of melanoma is available in Table 1.

## 4. Neurohormones and Melanoma

The term neurohormone is attributed to any substance produced by secreting cells within the nervous system and further released into the bloodstream, representing the connection between various stimuli and the chemical response resulting from their action.

### 4.1. The Corticotropin-Releasing Hormone–Proopiomelanocortin Axis

Hypothalamic hormones are involved in different processes such as growth, reproduction, lactation, metabolism, gastrointestinal function but also in stress response via the hypothalamic–pituitary axis. The skin is not just a target organ for corticotropin releasing hormone (CRH) and proopiomelanocortin (POMC)-derived neuropeptides but also a source of these peptides [154] and the CRH-POMC axis has been described as a main component of the skin neuroendocrine system [155,156].

Physiological and pathological factors, including various types of stressors, can in-fluence their skin expression levels [154,155,156]. Moreover, recent studies highlighted the patterns of hormone expression associated with the CRH-POMC axis in various skin tumors [157] and enzyme-linked immunosorbent assay (ELISA) has been used to assess the production of CRH, adrenocorticotropic hormone (ACTH), and alpha-melanocyte-stimulating hormone (α-MSH) in tumor cell lines and primary keratinocytes. However, the role of the CRH-POMC axis in skin tumors is largely unknown.

A significant difference between normal and tumor skin cells has been observed regarding the expression of hormones associated with the CRH-POMC axis. Immunohistochemical analysis of cutaneous tumors has shown that 80% of melanomas, 70% of squamous cell carcinomas, and 10% of basal cell carcinomas are highly immunoreactive for CRH [157]. Additionally, CRH together with CRH-R, are expressed in melanoma cell lines as well as in primary and metastatic melanoma. Furthermore, CRH expression is negative in benign nevus cells [158]. ACTH is strongly expressed in 70% of melanomas, 80% of squamous cell carcinomas, and 70% of basal cell carcinomas. Other POMC-derived peptides, such as α-MSH and melanocortin 1 receptor (MC1R) are also expressed in melanoma cell lines, nodular and metastatic melanoma. These results emphasize that skin malignancy is associated with an increased expression of neurohormones belonging to the CRH-POMC axis [157].

Moreover, the coordinated activity of the CRH-POMC axis was demonstrated in both primary and metastatic melanoma, colocalization of CRH and POMC peptides being emphasized in the great majority of CRH positive melanomas. Furthermore, in melanoma cell culture CRH induced the expression of POMC mRNA, while a CRH antagonist suppressed it [159].

CRH seems to be a significant mediator involved in the migration of melanoma cells. This process could be mediated through ERK1/2 signaling pathway and rely on interferences with the tumor microenvironment [160]. Another important possible effect of CRH is the reduction of tumor growth rate and was demonstrated in vivo on B16 melanoma cell lines. The suggested mechanisms were activation of endogenous CRH1 receptors and the alteration of intracellular Ca^2+^ signaling [161].

New research found that cellular adhesion molecule desmoglein 1 modulates the signaling between keratinocytes and melanocytes, with an increased production of POMC and cytokine mRNA, triggering increased melanin production and secretion. These effects are inhibited in the absence of UV exposure and might explain the pagetoid behavior of incipient melanomas [162].

Systemic administration of POMC was evaluated as melanoma therapy in mice, where it was evidenced that POMC overexpression prolonged survival. The administration of POMC determined melanogenic differentiation and reduced tumor growth by inducing apoptosis [163]. Moreover, it was shown that autophagy is involved in promoting cell death in POMC-mediated melanoma suppression through the α-MSH/hypoxia-inducible factor-1α/BNIP3/BNIP3L signaling pathway [164]. POMC also acts on the melanoma tumor microenvironment by reducing the neo-vascular network, thus demonstrating its potential role as a future treatment avenue [163].

### 4.2. Alpha-MSH

Alpha-MSH is created through the cleavage of the large peptide POMC and represents one of the most potent melanotropic substances. It can be produced by various cell types, including neural cells, endothelial cells, monocytes, and keratinocytes. Alpha-MSH is a hormone peptide consisting of the first 13 amino acids of the adrenocorticotropic hormone and its biological effects are mediated through melanocortin receptors, which are expressed in every skin cell type [165].

Alpha-MSH has an important role in the modulation of various skin inflammatory factors such as inflammatory cytokines, inflammatory transcription factors, adhesion molecules, and it also protects cutaneous cells from exogenous stress that can result after UV exposure or exposure to other inflammatory or oxidative stress inductive agents [165,166].

The increased cutaneous synthesis of α-MSH induced by UV exposure reduces the risk of skin cancer through several mechanisms, including reduction of hydrogen peroxide production, increased DNA repair capacity, maintaining genomic stability in melanocytes, and stimulation of melanogenesis. Moreover, α-MSH may also induce melanin production in melanoma cells impacting several pathways [167,168,169,170].

Studies have revealed that α-MSH can have an important role in melanoma, but its function is still unclear. Some studies have reported increased α-MSH plasma levels and expression in tumor tissue in patients with melanoma [171,172]. Another argument relies on the significant expression of α-MSH in over 50% of melanoma tumors [157]. Moreover, melanoma cells express functional cell-specific MSH receptors (MC1Rs) and the expression of these receptors seems to be independent of the cell cycle phase [173]. Different variants of the MC1R gene have been identified in normal skin and melanoma, and a deterministic connection between alleles of this gene and the susceptibility to melanoma has been suggested, but studies show controversial results [168,174,175].

Therefore, it is unclear whether α-MSH promotes or inhibits melanoma invasion or the immune response to melanoma.

Various in vivo studies have suggested that α-MSH acts as a melanoma inhibitor [176,177]. Moreover, in vitro and experimental studies have revealed that α-MSH may act on the tumor microenvironment, inhibiting the invasion capacity of melanoma cells and significantly reducing colony formation [177,178,179,180]. These effects can be induced by impacting several mechanisms. For example, α-MSH has inhibitory effects on proinflammatory factors, responsible for NF-κB activation which, in its turn, controls the expression of inflammatory cytokines and adhesion molecules [177,178,181]. Multiple studies have identified the ability of α-MSH to reduce the expression of adhesion molecules linked to the metastatic potential of melanoma cells [182] including ICAM-1 induced in melanoma cells by TNF-α [183,184]. It was revealed that ICAM-1 expression is higher in metastatic melanoma than in primary melanoma [185], thus decreasing the expression of ICAM-1 can be hypothesized to yield an antimetastatic effect. However, the ligands expressed by ICAM-1 on inflammatory cells may also serve the identification and subsequent removal of malignant cells [180,186]. Hence, there may be complex consequences of decreasing the expression of ICAM-1 in melanoma, and future studies are needed to clarify this issue.

It is also considered that α-MSH may delay the release of melanoma cells from the primary tumor (and initial metastasis), but on the other hand, can promote melanoma invasion by diminishing cell response to proinflammatory cytokines [187].

A significant biological function of α-MSH in melanocytes, probably also preserved in melanoma cells, is the shielding of melanocytic cells from proinflammatory cytokines and oxidative stress. A common response of cells to such stress includes the upregulation of adhesion molecules; some of these molecules bring cells to the attention of the immune system. Adhesion molecules such as integrins may also take part in the upregulation program and may promote melanoma interaction with extracellular matrix (ECM) proteins in terms of migration and invasion through ECM [187].

Studies revealed that while proinflammatory cytokine TNF-α increases the expression of integrins in HBL melanoma cells, α-MSH treatment leads to a reduction in integrin expression [188]. This suggests that α-MSH can effectively reduce cytokine-induced adhesion molecules such as integrins that would tend to increase melanoma invasion through ECM proteins and, at the same time, reduce the expression of adhesion molecules that promote the interaction of melanoma cells with the immune system [183,184], thereby increasing the escape of melanoma from immune control mechanisms.

These findings are supported by research revealing that uveal melanoma invasion is reduced by α-MSH and stimulated by TNF-α through a fibronectin-mediated mechanism [189].

There is also a diagnostic value of α-MSH in the management of melanoma which is worth mentioning. Labeling α-MSH with 99mTc offers high sensitivity for the diagnosis of melanoma, in both primary tumor and involved lymph nodes [190,191].

A melanoma-targeting MSH derivate with quick urinary clearance was recently proposed for evaluation of its potential in melanoma therapy. ^90^Y-DOTA-GGNle-CycMSH_hex_ shows good specific MC1R binding and retention in B16/F10 melanoma mice, but its clinical use is yet to be determined [192]. Additionally, ^18^F MSH derivates have successfully been used as PET imaging tracer substances for B16F10-luc melanoma in mice, with significant intratumor accumulation, clearly depicting small tumors [193,194]. Various ^64^Cu-, ^68^Ga-, and ^203^Pb-labeled, as well as 3 and 4-arm DOTA α-MSH analogs are also explored for their MC1R affinities [193,195,196,197,198].

UV-B radiation increases MSH production in melanoma cells in a dose-dependent manner. Moreover, UV-B exposure of melanoma cells increases the transcription of POMC mRNA, the production of POMC and ACTH, and also the expression of MSH receptors, most likely through a cAMP-dependent pathway [199].

However, alongside the previously discussed involvement in ICAM-1 expression, there are also other reports of carcinogenic effects of α-MSH mechanism [180]. Thus, α-MSH can reduce interactions between melanoma cells and T lymphocytes suggesting that it may assist melanoma cells in escaping from immune surveillance [200]. Alpha-MSH protects melanocytes and melanoma cells from the proinflammatory actions of TNF-α [180,201]. Therefore, α-MSH may have an immunomodulatory role that could prevent the immune recognition of melanoma cells (by an autocrine and/or paracrine mechanism) allowing metastatic transformation or dissemination [180,202,203,204].

### 4.3. Thyrotropin-Releasing Hormone

The tripeptide thyrotropin-releasing hormone (TRH) is considered to play a role in the human pigmentation process [205] and some studies outline similarities between MSH and TRH regarding the activation of the MC1R. TRH can bind to the MC1R expressed in B16 melanoma cell lines, where it stimulates the production of c-AMP [206]. Moreover, TRH and its receptor have long been identified both in melanoma cell lines [129,207,208] and in human tumor tissues [207,209]. Additionally, it is important to emphasize that low-concentration TRH administration induces an increase in melanoma cell proliferation, an effect that could not be highlighted in cell cultures of melanocytes [207]. In addition, TRH expression is more pronounced in melanomas and dysplastic nevi than in benign nevi, suggesting that TRH may be involved in the development of melanoma cells, possibly acting in a paracrine or autocrine manner [207].

### 4.4. Somatostatin

Somatostatin is a peptide hormone, involved in regulation of cell proliferation through its interaction with G protein-coupled somatostatin receptors (SSTRs). It is known as a potential antitumor agent and the expression of somatostatin receptors was emphasized in human melanoma tissue and melanoma cell lines [210]. However, in melanoma therapy, somatostatin analogs did not significantly inhibit melanoma evolution [211]. Nevertheless, a newly developed nanoparticle formulation of paclitaxel has demonstrated proapoptotic and invasion suppression effects on B16F10 mice melanoma cells that express somatostatin receptors, with no toxicity, while also inducing a favorable immune response through interactions with the tumor microenvironment [212]. Another somatostatin analog, pasireotide, has undergone Phase I study and the evaluation of its antitumor activity is pending [213].

The possible use of somatostatin receptors as molecular targets in anticancer therapy was also explored in uveal melanoma, which expresses mostly SSTR-2 and SSTR-5 [214]. While the use of somatostatin analogs was proposed as a potential therapy, their impact is undetermined, and there is no consensus regarding the role of SSTRs as prognostic markers in uveal melanoma [215,216].

### 4.5. Vasopressin

Vasopressin (antidiuretic hormone) is a nonapeptide neurohormone produced in the hypothalamic neurons and released by the neurohypophysis, with major roles in the hydroelectrolytic balance [217]. Moreover, it was described as an important modulator of socioaffective behaviors [218].

Investigating the possible expression of vasopressin in both melanoma cells and in normal human skin melanocytes, Aroni et al. [219] concluded that vasopressin does not play any role in the pathophysiology of this tumor.

However, desmopressin, a synthetic analog of the antidiuretic hormone, appears to influence melanoma evolution. Desmopressin treatment blocked the implantation of melanoma cells in different organs, exhibiting an important antitumor activity [220].

The effects of the aforementioned neurohormones in the biology of melanoma are synthetically presented in Table 2.

## 5. Neuropeptides and Melanoma

Neuropeptides are short sequences of amino acids linked together with peptide bonds. Their size varies from a few amino acids to structures that can contain over 50 amino acids [221]. There are numerous types of neuropeptides in the skin that can act on both neuronal and non-neuronal target cells. They can impact multiple intracellular effector systems related to cell proliferation or apoptosis, and are the subject of multiple cancer research studies worldwide [19,20,21,222].

### 5.1. Substance P

Substance P (SP) is a neuropeptide found in both the central and peripheral nervous systems. In the skin, it is expressed mainly in the sensory nerve fibers and acts through the neurokinin-1 receptor (NK-1R). SP induces local vasodilation, increased vascular, release of active compounds from mast cells (MC) as well as other proinflammatory effects [19,20,21,222].

The possible role of SP in melanoma is suggested by the increased expression of SP in lesions of in situ melanoma, primary invasive melanoma and metastatic melanoma, compared to benign nevi. Another interesting finding was that SP was strongly expressed also in dysplastic nevi [223]. Moreover, a study performed on canine melanoma tissues and cell lines found that the great majority of tumors express NK-1R immunoreactivity [224]. In another study, the NK-1 receptor has been identified in all investigated human melanoma tissue samples and cell lines and it was revealed that NK-1R plays an important role in the viability of melanoma cells [225].

SP demonstrates a great affinity for the tachykinin receptor NK-1R, and several studies have suggested that SP stimulates neo-angiogenesis, proliferation and invasion of melanoma cells via NK-1R [226,227,228,229,230]. This was further supported by results showing that human hemokinin-1, a highly selective NK-1R agonist, increases melanoma cell migration by promoting the expression of matrix metalloproteinases 2 and 14, which favor epithelial to mesenchymal transition as demonstrated also in other types of cancers [226,231].

Moreover, in vitro studies have shown that NK-1R antagonists such as Aprepitant can inhibit melanoma cell growth and induce apoptosis of tumor cells [223,227]. Another NK-1R antagonist labeled AA3266 has been recently identified as a strong inhibitor for melanoma MeW151 cell proliferation [232]. Another important finding was that the cancer growth inhibition induced by NK-1R antagonists depends on certain biochemical properties of the active substance, as the peptide Spandide I shows no antitumor activity on B16F10 melanoma cells, as opposed to the morpholine-based Aprepitant [233,234].

However, other studies have reported conflicting results regarding the effect of SP on melanoma. A study on B16F10 and B16LNAD melanoma cells has shown that SP inhibits tumor growth and potentiates the effects of radiotherapy [235].

Other research has suggested that the inhibitory effects of SP on melanoma can, at least partially, be mediated by the serotonergic system. Zhou et al. have demonstrated through in vitro experiments that SP can induce a direct apoptotic effect on B16F10 cells. This effect can be reduced by 5-HT and 5-HT_2A_ receptor agonists. Furthermore, SP inhibits the expression of the 5-HT_2A_ receptor on B16F10 murine melanoma cells. Thus, there is evidence regarding a bidirectional communication network between the SP/NK-1R and serotonergic systems which can be strongly involved in melanoma [123].

### 5.2. Calcitonin Gene-Related Peptide

Calcitonin gene-related peptide (CGRP) is a neuropeptide from the calcitonin family, with varied expression in different organs that seems to be influenced not only by humoral factors but also by direct contact of the secreting neuron with other cells [236].

CGRP can indirectly increase melanogenesis by stimulating keratinocytes to produce melanotrophic factors increasing the trophicity and melanin production of melanocytes [237].

However, other studies have revealed that CGRP working together with SP may have an opposite inhibitory effect on melanogenesis. Furthermore, CGRP could be able to induce apoptosis of melanocytes [238]. Regarding its relation to melanoma, Zhou et al. noted the proapoptotic impact of CGRP on B16F10 cells by increasing the Bax/Bcl-2 ratio [238].

### 5.3. Bradykinin

Another peptide possibly involved in skin cancer is bradykinin (BK). It is a nine-amino acids peptide chain that is produced by the kinin–kallikrein system through proteolytic cleavage of its kininogen precursors: high-molecular-weight kininogen and kallikrein. In the human body, BK is under the influence of angiotensin-converting enzyme, aminopeptidase, and carboxypeptidase [239]. BK produces vasodilation, reducing blood pressure, increases vascular permeability and is involved in pain mechanisms [240].

Different studies have suggested that BK can also be involved in modulation of cancer cell growth and tumor-associated angiogenesis via host stromal bradykinin B_2_ signaling [241]. Moreover, it can impact directly the tumor cells [242] by acting on two different subtypes of the kinin receptor, B_1_ and B_2_ [243,244,245,246]. Different studies have revealed that melanoma cells can express both of these receptors.

The effect of BK on melanoma was investigated in vitro on the B16-BL6 melanoma cell line. The study showed an increased expression and secretion of endothelin-1 by melanoma cells, probably induced by a direct effect of BK on B_2_ receptors [246]. This effect, along with endothelin-1 and bradykinin-mediated pain responses in mice orthotopically inoculated with melanoma cells, is inhibited by fentanyl citrate through μ-opioid receptors [247]. Activation of the host kinin B1 receptor leads to inhibition of melanoma growth and neo-angiogenesis, reduction of metastasis, and enhancement of the host immune response against disease progression, through combined action on melanoma cells and their tumoral milieu [248,249]. A recent study has investigated in a murine experimental model the role of the B_1_ receptor agonist des-Arg9-bradykinin (DABK) in melanoma metastasis. It has revealed that DABK-treated mice with melanoma had a lower number of tumor cells in their lungs, a lower expression of vascular cell adhesion molecule 1 (VCAM-1), and an enhanced CD8^+^ T-cell infiltration in the metastatic area when compared to those that did not get therapy. These results suggest a possible role of B_1_ receptor agonists in the management of metastatic melanoma [250].

### 5.4. Neuropeptide Y

Neuropeptide Y (NPY) consists of 36 amino acids and is part of the pancreatic polypeptide group. It is one of the most abundant brain peptides in the cortex, hippocampus, hindbrain, and hypothalamus, and also in the peripheral nervous system. It has different functions including cardiovascular modulation, stress response, neuroendocrine regulation, and stimulation of food intake [251].

Recent murine and human studies approached the possible association between neuropeptide Y and melanoma. A study published in 2012 by Gilaberte et al. reported NPY to be significantly expressed in melanomas, with a predominance in nodular type and with no correlation with other markers such as ulceration, mitotic index, or Breslow score [252]. On the other hand, Perez et al. outlined that NPY has a higher expression in superficial melanoma or lentigo maligna, while melanoma with low levels of NPY was characterized by intense cell proliferation, numerous peritumoral mast cells, and low expression of E-cadherin, concluding that high NPY expression in melanoma is associated with a better prognosis [253].

A study conducted on obese mice revealed that the use of NPY receptor blockers determines a decrease in tumor growth due to their effect on angiogenesis [254]. This discovery could prove useful in future melanoma therapies, although further studies are needed.

### 5.5. Galanin

Galanin is a neuropeptide consisting of 29/30 amino acids with wide-ranging effects. Even though it especially acts on the central and peripheral nervous systems, it also impacts other tissues and particularly the human skin. Both its endogenous and exogenous effects are mediated by three subtypes of receptors: GALR1, GALR2, and GALR3 [255].

Studies addressing the role of this neuropeptide in the skin showed that galanin exerts important anti-inflammatory functions, reduces vascular permeability and plasma extravasation, and modulates the vascular response associated with cutaneous neurogenic inflammation, counteracting the local effects of SP and CGRP [256,257,258]. Moreover, various studies have indicated galanin as a modulator of skin immune responses [257,258,259].

The involvement of galanin in several malignancies is well documented as well as its antiproliferative and proapoptotic activities revealed in various types of cancer, including epithelial tumors [260,261,262,263,264].

As regards the connection with melanoma, an observational retrospective study has evaluated the immunostaining for galanin in pigmented lesions, including primary and metastatic melanoma and various types of melanocytic nevi [265]. The expression of galanin was significantly higher in melanoma. However, there were differences depending on the tumor type, with the highest presence in superficial spreading melanoma. A very interesting aspect was the strong positive correlation found in melanoma samples between the expression of galanin and the immunostaining of another neuroendocrine factor, previously discussed in relation to melanoma—α-MSH [265].

Future studies assessing the expression of galanin receptors in different types of melanoma, the neuropeptide activity on melanoma cells in autocrine or paracrine pathways, and the possible interconnection with α-MSH are required. A thorough investigation of the link between galanin expression, cellular apoptosis, inflammation, and immune responses may help us to better define the role of this neuropeptide in melanoma.

### 5.6. Gastrin-Releasing Peptide

Gastrin-releasing peptide (GRP) is a neuroendocrine peptide with stimulating effects in the proliferation of certain types of cancer [266]. GRP was first identified in the enteric nervous system and data regarding its distribution in the skin is scarce. Studies in the field have demonstrated the immuno-expression of the GRP receptor (GRPR) in more than half of the melanoma cases included, albeit without any correlation to primary or secondary tumors or the Breslow index and Clark level [267]. However, elevated levels of GRP have been highlighted in nodular melanomas and melanomas with increased amounts of melanin [266]. Early studies have shown that A375-6 melanoma cell line growth is not hindered by bombesin, the GRP amphibian homolog, nor by GRP receptor antagonists [268].

However, Zhang et al. performed a study regarding the promising use of a specific protein fusion vaccine obtained from granulocyte macrophage colony-stimulating factor with GRP and gonadotrophin-releasing hormone. Their results outlined the inhibition of melanoma tumor growth in vivo, together with a decrease in tumor weight and volume [269].

### 5.7. Enkephalin

Enkephalins are endogenous opioids derived from pre-enkephalin. Structurally, they are pentapeptides, and two forms of enkephalin have been described, depending on their carboxy-terminal amino acids: methionine (met)-enkephalin (MENK) and leucine (leu)-enkephalin (LENK) [270]. Both of them are signaling through δ- and μ-opioid receptors (ORs), with a stronger affinity for the former [271]. These receptors of the opioid system are G protein-coupled receptors that also translate signals from other opioids [272,273]. These “classical” receptors were identified in keratinocytes, fibroblasts, and melanocytes, with a higher prevalence of δ-ORs [274]. Additionally, ORs have been identified in sebaceous and sudoriferous glands, hair follicles, nerve fibers, and immune cells in the skin [274]. However, another type of receptor called opioid growth factor receptor (OGFr), formerly known as ζ-receptor, has important properties, its stimulation by MENK causing inhibition of tumor cell proliferation, thus opening a new chapter in the research of the antitumoral potential of enkephalins [275].

Both forms of enkephalin, as well as proenkephalin (PENK), were isolated in keratinocytes, and their expression is decreased in melanocytic tumors compared to normal skin [276].

In terms of function, enkephalins exhibit a wide array of effects involving multiple systems. Enkephalins play major roles in analgesia, emotional behaviors, and stress response regulation [277,278]. However, of particular interest are studies that focused on their role in regulating cell proliferation.

MENK has shown antitumoral effects carried on through the OGFr in various malignancies, including pancreatic cancer, head and neck squamous cell carcinoma, ovarian cancer, neuroblastoma, colon cancer, and many others [279,280,281,282].

An early study investigating the effects of MENK on melanoma was performed by Murgo et al. on mice xenografts with B16-BL6 cells. The authors recorded growth restriction after the application of 50 µg of MENK for 1–2 weeks, which was countered by naloxone, confirming the opioid receptor involvement [283]; these effects were obtained via both direct cytotoxicity on melanoma cells as well as through immune response modulation. Using the same animal model, Wang et al. have identified that MENK augments OGFr expression, causes cell cycle arrest, and increases the ratio of CD4+/CD8+ T cells as well as plasma levels of various cytokines including IL-2, TNF-α, and interferon-γ [284]. These effects increase survival while limiting tumor growth and dissemination. In another study by the same research team, Wang et al. have concluded that MENK decreases the proliferation of A375 melanoma cells through similar mechanisms while also inducing apoptosis [285].

Imiquimod is a synthetic immune response modifier that exhibits antitumoral activity via the activation of toll-like receptor 7 and subsequent activation of NK cells, macrophages and lymphocytes that secrete various cytokines [286]. It was demonstrated that imiquimod upregulates the OGFr, thus amplifying the signaling axis between MENK and its receptor, augmenting the previously described antitumor properties [287]. Imiquimod has been successfully applied in the treatment of melanoma as presented in a large number of recent case reports [288,289,290,291]. Additionally, the topical use of imiquimod for melanoma cutaneous metastases has been well documented, concluding that it is useful in selected cases and is generally well tolerated [292].

The potential benefits of upregulating OGFr and using MENK in the management of melanoma demonstrated in in vitro studies are encouraging findings that are bound to attract further research interest in this class of substances and, ideally, the extension to human studies.

### 5.8. Beta-Endorphin

Beta-endorphin (β-endorphin) is an endogenous opioid peptide acting as an agonist for opioid receptors, with a high affinity for μ-OR, moderate affinity for δ-OR, and lower affinity for κ-OR [293,294]. Beta-endorphin is produced in neurons located in the hypothalamus, amygdala, and pituitary gland and plays multiple central roles in the organism, being involved in pain management, reward system, behavior, stress regulation, and other homeostatic functions [295,296,297,298]. However, β-endorphin also exhibits activity in peripheral tissues, most notably peripheral analgesia, through a mechanism involving an interplay with the immune system. In various inflammatory conditions, beta-endorphin released by T-cells activates ORs located on nerve endings thereby inhibiting the release of substance P and the reception of pain through sensory neurons [299,300].

These observations of interference between β-endorphin and the immune response have encouraged new studies towards exploring the potential use of β-endorphin as an immuno-modulator in cancer. Beta-endorphin was successful in reducing tumor growth in animal models of prostate and breast cancer by suppressing the activity of sympathetic neurons and stimulating parasympathetic activity, regulating peripheral immunity and cytokine levels [301,302,303]. However, a recent study shows that β-endorphin production is induced in mouse models of breast cancer and, in response, causes paradoxical increases in pain, tumor progression, and tumor survival [304].

There are also various studies regarding the possible role of β-endorphin in melanoma. In a very interesting study, Boehncke et al. showed that β-endorphin can be produced and released by B16 melanoma cells, and human melanoma biopsies have confirmed that β-endorphin expression is induced in these cells [305]. Moreover, it was revealed that β-endorphin liberated by melanoma cells and the μ-OR are deeply involved in modulation of tumor growth and tumor infiltrating immune cells, diminishing the host antitumor immune responses. Furthermore, the expression of β-endorphin in melanoma tissue is correlated with tumor progression [305].

There appears to be a concerted response of POMC, α-MSH, and β-endorphin in melanoma as the immunoreactivity of these peptides is higher and more diffuse in metastatic and advanced melanoma compared to benign melanocytic naevi, which confirms the involvement of the POMC gene and its derivated peptides [172]. This association was verified not only in melanoma but also in squamous cell carcinoma by using reversed-phase high-performance liquid chromatography on cell cultures [306].

The role of β-endorphin in the development of melanoma seems to be further emphasized by the observations that low-dose UV exposure induces the production of β-endorphin in epidermal keratinocytes, causing an increase in its plasma levels [307]. This causes an unfortunate unfavorable effect, as UV exposure was identified as biologically addictive, and β-endorphin may reinforce this conduct due to its implication in reward pathways, as Fell et al. have demonstrated on mice that exhibit this β-endorphin-related behavior [307]. Therefore, β-endorphin may act as an indirect causative factor in melanoma.

### 5.9. Vasoactive Intestinal Peptide

Vasoactive intestinal peptide (VIP) is a neuropeptide with wide distribution in the nervous system. However, it can be synthesized and released by other numerous cell types including immune and endocrine cells [308]. Its effect in melanoma cells could be of interest since VIP has been suggested to be involved in skin inflammation and modulation of skin immune responses [309].

An interesting study outlined the effect of VIP on B16F10 mouse melanoma cells, increasing microphthalmia-associated transcription factor (MITF) but also tyrosinase activity. Further investigations held on human cell lines suggested VIP-induced tyrosinase upregulation through MITF and CREB [309]. However, further studies are needed in order to explore in detail the impact of VIP on melanoma cells.

An overview of literature data describing the effects of neuropeptides in melanoma is available in Table 3.

## 6. Cellular and Molecular Neuro-Immune Interactions in Melanoma

In the skin, complex connections were emphasized between various nervous factors and different immune cells such as lymphocytes, macrophages, dendritic cells, neutrophils, and mast cells [19,310]. Moreover, an important role in stress-associated modulation of cutaneous inflammatory reactions is held by mast cells [311] which were also highlighted as potential players in different types of skin cancer [20,312,313]. The activity of the immune cells and their interactions are constantly modulated by various substances that mediate intra- and intercellular processes [314]. In this regard, nitric oxide holds particular interest, as various studies have suggested its potential role in melanoma pathogenesis.

### 6.1. Mast Cells

Mast cells are widely dispersed elements of the immune system responsible for a quick and sustained immune response [315]. Additionally, the proximity of mast cells to sensory nerves reveals another important role, which is the contribution to neurogenic inflammation triggered by substance P which induces mast cell degranulation. Upon stimulation, mast cells promptly release a variety of substances which include cytokines, chemokines, proteases, neuropeptides, growth factors, enzymes, and biological amines including histamine [316]. These factors have been identified as playing various roles in the development of melanoma, as further described in this section.

Mast cells are undoubtedly involved in melanoma, a claim supported by numerous findings of an increased density of mast cells around melanomas, in direct proportion to the aggressiveness of the tumor [317,318]. Moreover, the peritumoral number of mast cells also correlates with tumoral microvascularization, linking angiogenesis to the secretion products of mast cells [319]. Mast cells release a number of angiogenic factors, most notably VEGF, but also IL-8, heparin, and TGF-β; these substances are involved in the evolution of melanoma and correlated with its prognosis [65,320]. Furthermore, IL-8 also acts as a growth factor in melanoma, stimulating tumor growth in selected cell lines, SK-MEL 13 and SK-MEL 23, that produce IL-8 [321]. Basic fibroblast growth factor also exhibits this property of stimulating melanoma cell proliferation [322].

Besides angiogenesis, compounds resulting from mast cell degranulation can also enhance the immunosuppressive effect of UV-B exposure, a known carcinogenic agent for skin neoplasias [323]. The interference of these pathways is even more intricate, as it appears that UV-B may indirectly stimulate mast cells to release their contents by inducing keratinocyte-produced nerve growth factor and by activating a pathway resulting in increased release of SP and CGRP from sensory nerves in the skin [324,325,326]. In turn, mast cells release a range of active products the most important of which is histamine, a substance involved in local immune-inflammatory response that also triggers a cascade of cellular and humoral effects which were shown to decrease the efficiency of antitumoral defense mechanisms. This was emphasized by Nordlund et al. in a study on mice with Cloudman S91 melanoma where histamine promoted tumor growth while using histamine blockers inhibited cell proliferation [327]. 5-HT is also released by human mast cells after ionizing radiation exposure, having an inhibitory effect on melanoma development, reducing cell proliferation and improving fibronectin adhesion [328].

Further research in this area is needed, as mast cells appear to be involved in the mechanisms of melanoma development, through multiple pathways, some with divergent effects.

### 6.2. Nitric Oxide

Nitric oxide (NO) is a colorless gas, a free radical, and a signaling molecule that may be produced through various chemical reactions including the oxidation of L-arginine with NADPH with the participation of nitric oxide synthase (NOS) [329]. Three isoforms of NOS were described in the human body: neuronal (nNOS, NOS1), inducible (iNOS, NOS2), and endothelial (eNOS, NOS3) [330,331].

NO is a molecule with various biological functions, being involved in a wide range of physiological and pathophysiological processes. It is a multitasking player in the neuro-inflammatory mechanisms, with significant impact in neurotransmission, angiogenesis, inflammation, regulation of local blood flow, vascular permeability, leukocyte–endothelial interaction, platelet aggregation, and microlymphatic flow [331]. Its involvement in various malignancies was investigated and it was revealed that NO may contribute to tumor growth and metastasis by promoting the migratory, invasive, and angiogenic characteristics of tumor cells [331,332,333]. Furthermore, the expression of NO is dependent on the activity of the NOS isoforms, which have all been connected to the development and progression of various cancers [334].

In histological studies from melanoma patients, iNOS shows a higher expression in melanoma cells compared to melanocytes, in direct proportion to the Clark level, and was also identified in the periphery of lymph node metastases, as well as macrophages and blood vessel walls [329,335,336]

In addition to this molecule, the expression of nNOS was also demonstrated in primary melanomas [329]. Moreover, increased nNOS expression causes dysfunction of type I interferon in A375 melanoma cells, favoring lung metastasis [337]. The use of an nNOS inhibitor yielded a decrease in the intracellular level of NO and, consequently, a reduction of melanoma cell proliferation. Another aspect to be considered is that NO can be produced either by melanoma cell lines or by cells in the tumor microenvironment.

Moreover, eNOS was also correlated with melanoma development and metastasis, and also angiogenesis. A murine study on premalignant melanocytes by Melo et al. has found that eNOS is a key factor in the malignant transformation of these cells by promoting oxidative stress, therefore, favoring apoptosis and anoikis resistance [338]. The role of eNOS in angiogenesis was supported by the observation that stimulating VEGF receptors 2 and 3 induces eNOS activation in endothelial cells of lymph vessels with NO-mediated development and survival of these cells [339]. In the same study, Lahdenranta et al. also showed that eNOS mediates the production of new lymphatic vessels, favoring metastasis, which was demonstrated by the decreased lymph node metastasis rates in murine B16F10 melanoma models with eNOS genetic deletion. The correlation of eNOS with tumor angiogenesis was further emphasized by Barbieri et al. in mice with stress-promoted B16F10 melanoma, where a significant increase of VEGF was observed, which subsequently induced eNOS production [340]. This correlation was also identified in humans, on tissue biopsies of melanoma [341].

Regardless of the synthase that produces NO, it has been reported as presenting a dual effect in melanoma, both pro- and antitumoral. Shi et al. have shown that iNOS knockout mice demonstrate increased tumor growth in NO-sensitive melanoma cells but have lower metastasis rates in NO-resistant cells [342]. A recently initiated clinical trial aims to phenotype subsets of immune cells to detect NO levels before and after treatment, as it appears that NO can modulate the activity of specific immune cells, impacting relapse-free survival [343]. Conversely, in a study on Lu1205 metastatic melanoma cells, Yang et al. showed that NO induces human apurinic (apyrimidinic) endonuclease/redox-factor 1 resulting in a feedback loop that favors the growth and dissemination of melanoma cells [344]. Additionally, a study on a murine B16 melanoma model has shown that NO mediates endothelial cell differentiation, as well as blood vessel development and growth [345]. Similar findings were identified on A375 melanoma cells where VEGF-induced NO production stimulated tumor cell proliferation [346].

NO has been successfully used as a mediator in overcoming melanoma resistance to doxorubicin. Thus, photoregulated NO release from nanoparticles acts as a facilitator for doxorubicin, increasing cellular retention and amplifying its anticancer effects [347]. NO also enhances the antitumor activity of Ritonavir on primary and metastatic melanomas, by permanently inhibiting S6 protein and regulating p53 expression [348]. Adding a NO moiety to lopinavir doubles the anticancer activity in vitro on B16 cells [349].

NO-releasing STAT3 inhibitors showed antiproliferative activity in A375 melanoma cells carrying the BRAFV600E mutation via reactive oxygen species production and G1 cell cycle arrest [350]. Other NO-releasing agents have also been researched, acting as a successful anti-inflammatory and antitumoral agents, both in vivo and in vitro [351].

A summary of evidence regarding the involvement of NO and its synthases in melanoma is presented in Table 4.

## 7. Discussion

Research conducted in recent years has led to the conclusion that in the appearance and development of melanoma, in addition to the already established risk factors such as ultraviolet radiation, fair skin, thinning of the ozone layer due to pollution, the presence of multiple, atypical or congenital pigmented nevi, familial history of melanoma, and immunosuppression, it is necessary to take into account the neuroendocrine component and its role in this pathology. Recent studies on this topic, including several clinical trials, have explored the interferences of various drugs with melanoma biology by modulating the neuroendocrine axis (Table 5). While some of the results are promising, others have not met the expectations, and continued research will undoubtedly clarify the extent to which pharmaceutical regulation of neuroendocrine factors can demonstrate benefits in melanoma patients.

Although acute stress appears to have a protective role in carcinogenesis, chronic stress determines the release of different neuropeptides, neurohormones, and cytokines that cause immunosuppression and participate in the induction and maintenance of the carcinogenesis process. This is of paramount importance as chronic stress has become an increasingly significant part of everyday life. Further research is warranted in the majority of neoplasias, not only in melanoma. Neurotransmitters like catecholamines, glutamate, serotonin, cannabinoids, as well as neurohormones such as CRH, POMC, α-MSH, TRH, somatostatin, vassopressin and neuropeptides including SP, CGRP, bradykinin, neuropeptide Y, galanin, GRP, enkephalin, β-endorphin, and VIP should be taken into consideration even though their role is still debated. Mast cells capable of releasing multiple substances that potentially modulate tumor evolution, as well as small signaling molecules such as NO are also key factors in melanoma biology. Additionally, all this gathered information could potentially open new treatment avenues and would, ideally, be used in the development of novel therapeutic protocols that can lead to increased survival rates.

## Figures and Tables

**Table 1:** Summary of the effects of neurotransmitters on melanoma cells/tumors.

Factor	Experimental Model	Mechanism	Effect	Reference
Catecholamines				
Norepinephrine	In vitro (C8161, 1174MEL, and Me18105)	Release of VEGF, IL-6, IL-8	Stimulation of melanoma aggressiveness	[38]
	In vitro (A375 cells)	Activation of β3-ARs	Recruitment of stromal cells	[73]
Norepinephrine, epinephrine	In vitro (B16F10 melanoma cells)	β-Ars ^1^-mediated effects	Stimulation of cell proliferation	[17]
	In vitro (Hs29-4T and A375 cells)	Activation of MAPKs and MMPs 2 and 9	Stimulation of motility and invasion	[58]
Epinephrine	In vitro (FM-55-P, 92-1, Mel202, and A375 cells)	β2-ARs-mediated effects	Stimulation of cell proliferation and invasion	[59]
Epinephrine	In vivo (mice with B16F10 melanoma inoculations)	Mobilization of NK cells and redistribution to tumors in regular exercise	Inhibition of tumor incidence and growth	[96]
Phenylephrine	In vitro (SK-Mel 23 cells)	p38 and ERKs signaling via α1-ARs stimulation	Inhibition of cell proliferation	[97]
Glutamate				
Glutamate	In vivo (mice with C8161 xenografts)	Activation of mGlut1	Stimulation of cell proliferation and metabolic activity	[105]
	In vivo (mice with UACC903-G4 xenografts)	Activation of PI3K/Akt/mTOR pathway	Stimulation of angiogenesis in xenografts	[110]
Serotonin and Analogs				
Serotonin	In vitro (B16F10 melanoma cells)	5-HT_2A_-mediated cross-talk with SP	Neutralization of the apoptotic effects of SP	[123]
Serotonin	In vitro (B16F10, SK-MEL-2, and Melan-A cells)	5-HT_2A_-mediated increase in tyrosinase activity, dendritic network, and melanin production	Induction of melanogenesis	[124]
1-NPZ ^2^	In vitro (MNT-1 melanoma cells)	Increased expression of Cox-2 and ROS production.	Induction of apoptosis	[130]
NCS, NPCS ^3^	In vitro (murine B16 and human HMV-II melanoma cells)	Inhibition of tyrosinase	Suppression of melanogenesis	[131]
Tegaserod	In vivo (mice with B16F10 melanoma inoculations)	Inhibition of PI3K/Akt/mTOR pathway	Inhibition of tumor growth and dissemination. Increase in survival	[134]
Cannabinoids				
Anandamide	In vitro (A375 melanoma cells)	CB1-mediated induction of caspase-dependent apoptosis; Cox-2 and Lox-mediated cytotoxicity	Induction of apoptosis	[143]
THC, WIN 55,212-2	In vitro (B16 and A375 melanoma cells)	CB1/2-mediated inhibition of Akt signaling inhibition	Induction of apoptosis. Inhibition of cell proliferation and metastasis	[145]
Anandamide, ACEA ^4^	In vitro (HT168-M1 and WM983B melanoma cells)	CB1-mediated inhibition of PI3K/Akt/mTOR pathway	Inhibition of cell proliferation and metastasis	[147]
Cannabidiol	In vivo (mice with B16F10 melanoma inoculations)	Possibly CB2-mediated antitumoral activity	Inhibition of tumor growth. Increased survival and quality of life	[149]
WIN 55,212-2	In vitro (COLO38, SKMEL28, and OCM1A melanoma cells)	Lipid raft-mediated activation of capsase-9 and ERK pathways	Inhibition of cell proliferation	[150]
THC, Cannabidiol	In vitro (CHL-1, A375, and SK-MEL-28 melanoma cells)	Atg7-mediated autophagy; TRIB3-mediated apoptosis	Inhibition of cell viability	[151]
*Cannabis*	Human retrospective study	Untested, possibly PD-1 or PD-L1 inhibition	Decrease of immunotherapy (nivolumab) response rate	[153]

^1^ ARs = adrenergic receptors; ^2^ 1-NPZ = 1-(1-Naphthyl) piperazine; ^3^ NCS = N-caffeoylserotonin, NPCS = N-protocatechuoylserotonin; ^4^ ACEA = Arachidonyl-2-chloroethylamide.

**Table 2:** Summary of the effects of neurohormones on melanoma cells/tumors.

Factor	Experimental Model	Mechanism	Effect	Reference
CRH-POMC				
CRH	In vitro (B16F0 and B16F10)	Activation of ERK1/2 pathways	Stimulation of tumor cell migration	[160]
CRH and various analogs	In vitro (murine Cloudman and B16 tumors)	CRH1-mediated Ca^2+^ signaling alteration	Inhibition of cell proliferation	[161]
POMC	In vivo (mice with B16F10 melanoma inoculations)	Induction of differentiation. Direct effect on endothelial cells.	Inhibition of cell proliferation and angiogenesis. Increased survival	[163]
		Autophagy and apoptosis via α-MSH/HIF-1α/ BNIP3 signaling pathway	Induction of apoptosis	[164]
Alpha-MSH				
α-MSH	In vitro (B16BL6 and B16F1 cells)	Inhibition of MMPs 2 and 9	Inhibition of invasion and migration	[179]
	In vitro (A-375SM and HBL melanoma cells)	Reduction of NF-κB DNA activation	Immunomodulation. Inhibition of proinflammatory cytokine effects	[180]
	In vitro (STG, RIDE, WR2 cultures, HBL, DOR cells)	MC1R-mediated reduction of ICAM-1 expression	Inhibition of proinflammatory cytokine effects	[183,184]
	In vitro (HBL and A375-SM melanoma cells)	MC1R-mediated elevation of cAMP and intracellular Ca^2+^	Inhibition of invasion	[187]
	In vitro (HBL melanoma cells)	Decreased integrin expression	Inhibition of invasion	[188]
	In vitro (SOM, 177w7B7, and VUP cell lines)	Modulation of tumor microenvironment. Opposing effects to TNF-α	Inhibition of invasion	[189]
	In vitro (A375-SM and HBL cells)	Decrease in B7 expression	Assists melanoma cells in immune evasion	[200]
		Inhibition of NF-κB binding activity	Assists melanoma cells in immune evasion	[201]
TRH				
TRH	In vitro (COS and B16 melanoma cells)	Stimulation of cAMP production	Similar MC1R activation to α-MSH	[206]
Somatostatin Analogs				
Octreotide and SOM230	In vitro (18 human melanoma cell lines)	Receptor-mediated mechanism (mainly SSTR-2)	Inhibition of cell proliferation	[211]
PSM ^1^	In vivo (mice with B16F10 melanoma inoculations)	Calreticulin exposure. Decreased expression of Ki67. Modulation of EMT markers expression. Increased TNF-α, IFN-γ, and IL-2 in T cells	Induction of apoptosis. Inhibition of cell proliferation and invasion	[212]
Pasireotide	Patients with advanced melanoma (n = 10)	Inhibition of Ki-67 expression and serum levels of IGF-1, IGF-2, MIA, S100B, and IGFBP3	Partial response (n = 1)/stable disease (n = 1)	[213]
Vasopressin Analogs				
Desmopressin	In vivo (mice with B16F10 melanoma inoculations)	TIMP-1-dependent prevention of melanoma cells implantation	Inhibition of dissemination	[220]

^1^ PSM = paclitaxel-based lipid nanoparticles with Tyr-3-octreotide.

**Table 3:** Summary of the effects of neuropeptides on melanoma cells/tumors.

Factor	Experimental Model	Mechanism	Effect	Reference
Substance P/NK-1R Antagonists				
Substance P	In vitro (B16F10 cells)	NK-1R-mediated inhibition of MAPK and decrease in tyrosinase activity	Inhibition of melanogenesis	[234]
Maropitant	In vitro, in vivo (canine melanoma tissue/cells)	Possible NK-1R-mediated effects	Inhibition of cell proliferation. Induction of apoptosis	[224]
Aprepitant	In vitro (COLO 858, MEL HO, and COLO 679 cells)	Inhibition of SP-induced mitogen stimulation	Inhibition of cell proliferation	[225]
L732138	In vitro (A375 and B16F10 cells)	Inhibition of MMP-2 and -9 expression via ERK1/2, JNK, and p38 signaling	Inhibition of dissemination	[226]
L-733 060	In vitro (COLO 858, MEL HO, and COLO 679 cells)	Inhibition of mitogenesis via MAPK pathway	Inhibition of cell proliferation	[228]
Cyclosporin A	In vitro (COLO 858, MEL HO, and COLO 679 cells)	Inhibition of SP-induced mitogen stimulation	Inhibition of cell proliferation	[229]
AA3266	In vitro (MeW164, MeW155, MeW151 cells)	Decreased Ki-67 expression. Induction of cell cycle arrest	Inhibition of cell proliferation	[232]
CGRP				
CGRP	In vitro (B16F10 cells)	Suppression of NF-κB activation. Promotion of apoptosis	Inhibition of cell proliferation	[238]
Bradykinin				
Bradykinin	In vitro (B16-BL6 cells)	B2-mediated increased expression of endothelin-1	Stimulation of cell proliferation	[246]
DABK	In vivo (murine Tm5 melanoma)	B1-mediated increase in Ca^2+^ concentration and ERK phosphorylation	Inhibition of cell proliferation, invasion, dissemination, and vascularization	[249,250]
Neuropeptide Y Analogs				
BIIE 0246	In vivo (mice with B16F10 melanoma inoculations)	NPY Y2-R antagonism. Decrease in VEGF serum levels	Inhibition of cell proliferation and angiogenesis	[254]
GRP				
GRP	In vivo (mice with B16F10 melanoma inoculations)	Antibodies induced via a multicomponent vaccine formulation	Inhibition of cell proliferation	[269]
Enkephalins				
MENK	In vivo (mice with B16F10 melanoma inoculations)	Stimulation of host immune response. Direct cytotoxicity. Modulation of tumoral milieu	Inhibition of cell proliferation	[283]
		Induction of cell cycle arrest. Increased TNF-α, IFN-γ, and IL-2	Inhibition of cell proliferation. Increased survival	[284]
	In vitro (A375 cells)	Induction of cell cycle arrest. Decreased expression of survivin	Inhibition of cell proliferation. Induction of apoptosis	[285]
Beta-Endorphin				
β-endorphin	In vivo (mice with B16F10 melanoma inoculations)	Inhibition of tumor infiltration by lymphocytes	Stimulates cell proliferation. Inhibition of the host immune response	[305]
Vasoactive Intestinal Peptide				
VIP	In vitro (B16F10 cells)	PKA-CREB-mediated increase in tyrosinase activity	Induction of melanogenesis	[309]

**Table 4:** Summary of the effects of neuroendocrine factors on melanoma cells/tumor.

Factor	Experimental Model	Mechanism	Effect	Reference
iNOS	Ex vivo (various human melanoma tissue specimens)	Stimulation of blood and lymph angiogenesis	Stimulation of blood and lymphatic dissemination through angiogenesis	[335]
	In vivo (mice with B16BL6 melanoma inoculations)	Modulation of NO-sensitive macrophages’ activity	Stimulation of cell proliferation and dissemination	[342]
nNOS	In vitro (A375 cells) In vivo (mice with B16F10 melanoma inoculations)	Inhibition of IFNα signaling and tumor infiltration by lymphocytes	Stimulation of dissemination	[337]
uncoupled eNOS	In vitro (Tm5 melanoma cells)	Production of superoxide	Stimulation of cell proliferation and malignant transformation	[338]
eNOS	In vivo (mice with B16F10 melanoma inoculations)	Stimulation of peritumor lymphatic hyperplasia via PI3K pathway activation	Stimulation of cell proliferation and dissemination (lymphatic)	[339]
		β-adrenergic stimulation of cAMP-PKA signaling	Stimulation of cell proliferation in stress conditions	[340]
	Ex vivo (melanoma tissue sections)	VEGF-mediated increase in microvascular density	Stimulation of angiogenesis	[341]
NO	Ex vivo (advanced melanoma resections)	Modulation of immune cells’ activity	Increased/decreased survival dependent on NO source	[343]
	In vitro (Lu1205 metastatic melanoma cells)	APE/Ref-1-mediated activation of oncogenic targets	Stimulation of cell proliferation and dissemination	[344]
	In vivo (mice with B16F1 and B16F10 inoculations)	Direct effect on endothelial cells. Indirect effect by stimulating angiogenic factors	Stimulation of angiogenesis and dissemination	[345]
	In vitro (A375 cells)	VEGF-induced stimulation of iNOS expression	Stimulation of cell proliferation	[346]
	In vitro (M14 DOX-resistant melanoma cells)	NO delivered by nanoparticles increases DOX retention	Stimulation of the antitumoral activity of doxorubicin	[347]
	In vitro (metastatic B16F10 cells)	Induction of senescence. Inhibition of S6 protein	Stimulation of the antitumoral activity of Ritonavir	[348]
	In vitro (B16, B16F10, A375 cells) In vivo (mice with B16 melanoma inoculations)	Production of ROS and RNS. Inhibition of P70S6K	Stimulation of the antitumoral activity of Lopinavir	[349]
	In vitro (A375 cells)	NO delivered by synthetic quinolone donors. Inhibition of STAT3 tyrosine phosphorylation. Production of ROS. Induction of cell cycle arrest	Inhibition of cell proliferation	[350]
	In vivo (mice with B16F10 melanoma inoculations)	NO delivered by triptolide/furoxans hybrids	Inhibition of cell proliferation. Anti-inflammatory effects	[351]

**Table 5:** Recapitulation of recent clinical trials reporting effects on the neuroendocrine axis of melanoma.

Tested Substance	Study Type	Mechanism	Effect	Reference
β-blockers (metoprolol, propranolol, atenolol, and others)	Cohort study (4179 patients)	Blocking βARs	Increased survival in melanoma patients	[83]
β-blockers (unspecified)	Cohort study (121 patients)	Blocking βARs	Reduced risk of disease progression	[84]
β-blockers (pan or β1 selective)	Cohort study (195 patients)	Inhibition of stress signaling, particularly via β2AR signaling	Increased survival in melanoma patients	[86]
β-blockers (pan or β1 selective)	Cohort study (203 patients)	Blocking βARs	No impact on survival in melanoma patients	[91]
Riluzole	Phase 0 trial (12 patients)	Glutamate blockade inhibiting MAPK and PI3k/Akt signaling	Melanoma metabolic activity suppression was achieved. Inconsistent effects	[117]
Riluzole	Phase II trial (13 patients)	Inhibition of GRM1 signaling Increased leukocyte infiltration	Antitumoral biological effects. No tumoral response recorded according to staging criteria	[100]
Ipilimumab	Phase II trial (75 patients)	NO-mediated modulation of the tumor microenvironment	Mixed anti- and pro-melanoma activity. Inconsistent effects of NO and metabolites	[343]
SSRIs (unspecified)	Cohort study (5591 patients)	Inhibition of serotonin uptake	Decrease in survival of melanoma patients	[141]
*Cannabis*	Cohort study (140 patients)	Complex interaction with the immune response, possibly CB2-mediated	Decrease of response rate to nivolumab in patients with advanced melanoma	[153]
Pasireotide	Phase I trial (10 patients)	SSTRs-mediated Ras/MAPK signaling modulation	Stable disease and partial response were obtained. Progression of disease also recorded in some patients	[213]
